# Characterization of novel Omp31 antigenic epitopes of *Brucella melitensis* by monoclonal antibodies

**DOI:** 10.1186/s12866-017-1025-3

**Published:** 2017-05-15

**Authors:** Jinfeng Li, Feihuan Hu, Shouyi Chen, Peifang Luo, Zuoping He, Wenjing Wang, Jean-Pierre Allain, Chengyao Li

**Affiliations:** 1 Department of Transfusion Medicine, Southern Medical University, Guangzhou, China; 2 Guangzhou Center of Disease Control and Prevention (CDC), Guangzhou, China; 3 Qingyuan People’s Hospital, Qingyuan, Guangdong China; 4 Department of Hematology, University of Cambridge, Cambridge, UK; 5 School of Public Health and Tropical Medicine, Southern Medical University, Guangzhou, China

**Keywords:** *Brucella melitensis*, Omp31 antigen, Monoclonal antibody, Epitope identification, Conserved epitope

## Abstract

**Background:**

Brucellosis is a severe zoonotic disease worldwide. Detection and identification of *Brucella* species are essential to prevent or treat brucellosis in humans and animals. The outer membrane protein-31 (Omp31) is a major protein of *Brucellae* except for *B. abortus,* while the Omp31 antigenic epitopes have not been extensively characterized yet*.*

**Results:**

A total of 22 monoclonal antibodies (mAbs) were produced against Omp31 of *Brucella* (*B.*) *melitensis*, of which 13 recognized five linear epitopes, 7 reacted with semi-conformational epitopes and 2 reacted with conformational epitopes, respectively. The mAb isotypes were 11 (50%) IgG2a, 5 (23%) IgG1 and 6 (27%) IgM. On the basis of epitope recognition and reactivity levels, 8 mAbs including 3 IgM and 5 IgG clones were considered as highly reactive and potentially diagnostic antibodies. Among these mAbs, 7A3 (IgG1), 5B1 (IgG2a), 2C1 (IgG2a) and 5B3 (IgG2a) reacted with differently conserved linear epitopes of *B. melitensis*, *B. ovis*, *B. suis* and *B. canis* strains, while 5H3 (IgG2a) highly reacted with a conformational epitope of Omp31 when tested with several immunoassays.

**Conclusions:**

These potent monoclonal antibodies can be used for identifying Omp31 antigens or detecting *B. melitensis* and other *Brucella* species beyond *B. abortus* in vitro or in vivo.

## Background

Brucellosis is one of the most serious zoonoses worldwide. It causes severe diseases in humans and substantial animal losses. In China, human brucellosis cases are increasing. In 2015, 56,989 new cases of human brucellosis have been reported according to Chinese CDC (http://www.chinacdc.cn/), of them are mostly caused by *Brucella* (*B.*) *melitensis* infected sheep and goats or their products [1–3].

The 31–34 kDa outer membrane protein (OMP) or (Omp31) is a major membrane protein of *Brucellae* except for *B. abortus* [4]. Omp31 plays an important role in cellular and humoral immune protective responses against *Brucella* infection [4–11]. Previously we fully evaluated Omp31 epitopes in specific T-cell response in sheep vaccinated with attenuated *B. melitensis* vaccine [12]. However, the B-cell epitopes have not yet been extensively investigated. To date, only few epitopes recognized by antibodies to *Brucella* Omp31, such as monoclonal antibody A59/10F09/G10 recognizing amino acid 48–83 of *B. melitensis* M16 and presenting protective activity were reported [4, 13, 14]. In this study, we generated and characterized 22 novel murine monoclonal antibodies (mAbs) binding native Omp31 of *B. melitensis*. Some of these antibodies presented high reactivity with different epitopes of Omp31, suggesting potential capacity to identify Omp31 antigens of *Brucellae* or to detect *B. melitensis or* other *Brucella* species beyond *B. abortus*.

## Results

### Production of mAbs to Omp31 of *B*. *melitensis*

A total of 22 mAbs reactive with recombinant Omp31 (rOmp31) were selected from screening of hybridomas by EIA (Table 1). Antibody isotypes were identified for all 22 mAbs, including 50% (11/22) IgG2a, 23% (5/22) IgG1 and 27% (6/22) IgM, respectively.

**Table 1:** Characterization of mAbs reactive to the epitopes within Omp31 of *B*. *melitensis*

MAb	Isotype	Peptide ELISA	rOMP31 Western-Blot	NMP Western-Blot	NMP ELISA	SSP ELISA	TC IFS	*B. m* ICS	Epitope (aa)/peptide
1H2	IgG1 (K)	12	++	−	<1	1	+	−	L (33–48/42–57)/P05/06
2D2	IgM (K)	7	++	++	4.5	4.8	+	++	L (33–48/42–57)/P05/06
2G9	IgG1 (K)	3	++	++	<1	2.2	+	+	L (33–48/42–57)/P05/06
7A3	IgG1 (K)	1.2	++	++	7	17	+	+	L (33–48/42–57)/P05/06
5B1	IgG2a (K)	3.8	+	−	1.2	2	+	++	L (87–102)/P11
2C1	IgG2a (K)	2.7	++	++	2.4	5.5	++	++	L (159–174/168–183)/P19/20
2E7	IgG2a (K)	1.8	++	++	1.5	3.5	++	±	L (159–174/168–183)/P19/20
4E9	IgG2a (K)	3.7	+	++	2	5	++	++	L (159–174/168–183)/P19/20
4H10	IgG2a (K)	1.9	++	++	2.2	4.2	++	−	L (159–174/168–183)/P19/20
8F11	IgG2a (K)	2.2	++	++	2.5	5.5	++	+	L (159–174/168–183)/P19/20
2A8	IgG1 (K)	7.8	++	+	<1	1.5	(+)	+	L (177–192)/P21
6D8	IgG1 (K)	4.7	++	−	<1	<1	+	±	L (177–192)/P21
5B3	IgG2a (K)	2.8	++	+	<1	16	+	++	L (204–219)/P24
2B6	IgM (K)	<1	++	−	8	11	−	+	SC(20–240)
4H6	IgM (K)	<1	++	−	<1	1.2	−	±	SC(20–240)
5B11	IgM (K)	<1	++	−	5.8	9.2	−	±	SC(20–240)
5H3	IgG2a (K)	<1	++	++	11	18	+	++	SC(20–240)
6E10	IgG2a (K)	<1	+	+	1	2	++	−	SC(20–240)
6F9	IgG2a (K)	<1	+	++	<1	1.5	++	±	SC(20–240)
11C4	IgG2a (K)	<1	++	++	1.2	2.6	++	±	SC(20–240)
2H5	IgM (K)	<1	−	−	4.1	7	−	−	C (20–240)
5F11	IgM (K)	<1	−	−	8.2	12	−	+	C (20–240)

NMP, native membrane proteins extracts of *B*. *melitensis*; SSP, supernatant of sonicated *B*. *melitensis* proteins; TC IFS, Lentivirus-mediated Omp31, transduced 293Tcells detected by immunofluorescent staining; *B.m* ICS, *B*. *melitensis* strain detected by immunochemical staining; L, linear; SC, semi-conformational; C, conformational. The numbers indicate the S/CO values in the EIA; the reactivity levels are indicated by ++ (strongly reactive), + (reactive), ± (indeterminate) or – (non-reactive) in the Western-blot, IFS and ICS. (+), indicate false positive for the negative control of cells. Five IgG and three IgM clones in bold are representatives of high capacity of mAbs reacting with native Omp31 antigens of *B*. *melitensis* in various assays.

### Classification of Omp31 epitopes by mAb’s recognition

**Fig. 1:**
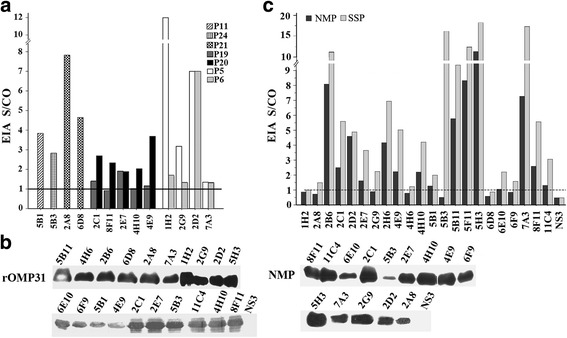
Reactivity of mAbs to Omp31 antigens of *B*. *melitensis*. (a) Binding of mAbs to 16mer overlapping peptides derived from Omp31 in Peptide-ELISA. (**b**) Identification of mAbs reacting with rOmp31 and NMP (native membrane protein extracts of *B. melitensis* M5–90) by Western-blot. (**C**) Reactivity of mAbs to NMP and SSP (supernatant of sonicated proteins of *B. melitensis* M5–90) by ELISA. NS3, an HCV NS3 peptide, recombinant protein and an un-related mAb to HCV NS3 were used as negative-controls, respectively. The dotted line indicates the level of cut off defined as mean + 2SD of OD value to negative controls

In order to classify the Omp31 antigenic epitopes, all mAbs were tested for reactivity with 27 16mer overlapping peptides derived from full-length amino acid (aa) sequence, denatured or non-denatured protein forms of *B*. *melitensis* Omp31 in various immunoassays. Thirteen mAbs were reactive with 7 linear peptides in Peptide-ELISA (Fig.1a). Twenty mAbs were reactive to the denatured rOmp31 and 14 mAbs to the denatured native membrane protein extract (NMP) by Western blot (Fig. 1b), respectively. The mAbs reactivity was also tested against the non-denatured native antigens in ELISA using the NMP or the supernatant of sonicated proteins (SSP) from *B*. *melitensis*, which showed that 16 and 20 mAbs were reactive, respectively (Fig. 1c).

According to the nature of Omp31 antigens recognized by 22 mAbs, the epitopes were stratified into three groups of linear (L), semi-conformational (SC) and conformational (C) forms. Among these 22 mAbs, 13 reacted with the linear epitopes, 7 reacted with the semi-conformational and 2 reacted with the conformational epitopes presented in either rOmp31 or native Omp31 antigens of *B*. *melitensis* (Table 1).

### Linear epitope mapping of Omp31 by mAbs

**Fig. 2:**
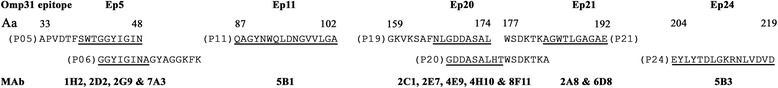
Mapping for linear epitopes of Omp31 recognized by mAbs. The amino acid (aa) sequences of 16mer peptides reactive to the mAbs are presented, of which the epitopes (Ep) are designated on the top of underlined aa sequences. Aa position of Omp31 is indicated at the beginning and the end of the peptide sequence. MAbs are indicated below the epitopes they recognize

**Fig. 3:**
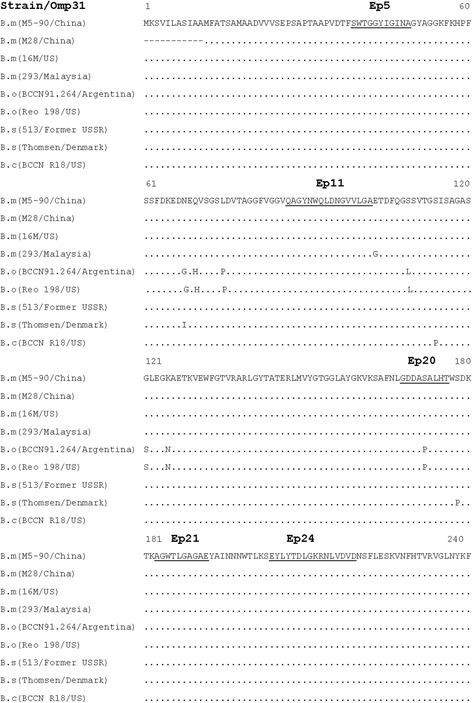
Alignment of Omp31 sequences from four species of *Brucella* strains. The aa sequences of Omp31 from *B. melitensis* (B.m), *B. ovis* (B.o), *B. suis* (B.s) and *B. canis* (B.c) strains were retrieved from Genbank database. The accession numbers are ADZ88512.1 (M5–90/China), ADZ67646.1 (M28/China), P0A3U4.1 (M16/US), ACQ84164.1 (293/Malaysia), AAL27294.1 (BCCN 91.264/ Argentina), AAL27292.1 (Reo 198/US), AAL27290.1 (513/Former USSR), AAL27287.1 (Thomsen/Denmark), AAL27296.1 (BCCN R18/US). The identified mAb’s recognition epitopes are underlined on top of aa sequences

Among seven reactive linear peptides (Fig. 1a), the epitope shared by peptides P05 and P06 was reactive with mAbs 1H2, 2D2, 2G9 and 7A3. However, due to the stronger reactivity with P05 than P06, the minimal aa common sequence of Omp31 was designated as epitope Ep5 (^39^SWTGGYIGINA^49^) (Fig. 2). Similarly, epitope Ep20 (^168^GDDASALHT^176^) overlapped by peptides P19 and P20 reacted with mAbs 2C1, 2E7, 4E9, 4H10 and 8F11. Epitope Ep21 (^183^AGWTLGAGAE^192^) reacted with both mAbs 2A8 and 6D8. Epitopes Ep11 (^87^QAGYNWQLDNGVVLGA^102^) and Ep24 (^204^EYLYTDLGKRNLVDVD^219^) were recognized only by mAb 5B1 or 5B3, respectively (Fig. 2). Alignment of Omp31 aa sequences showed that these five epitopes were completely conserved among *B*. *melitensis*, *B. ovis*, *B. suis* and *B. canis* except for a single aa mutation (S172P) within Ep20 of *B. ovis* strains (Fig. 3).

### Recognition of Omp31-lentivirus transduced cells

**Fig. 4:**
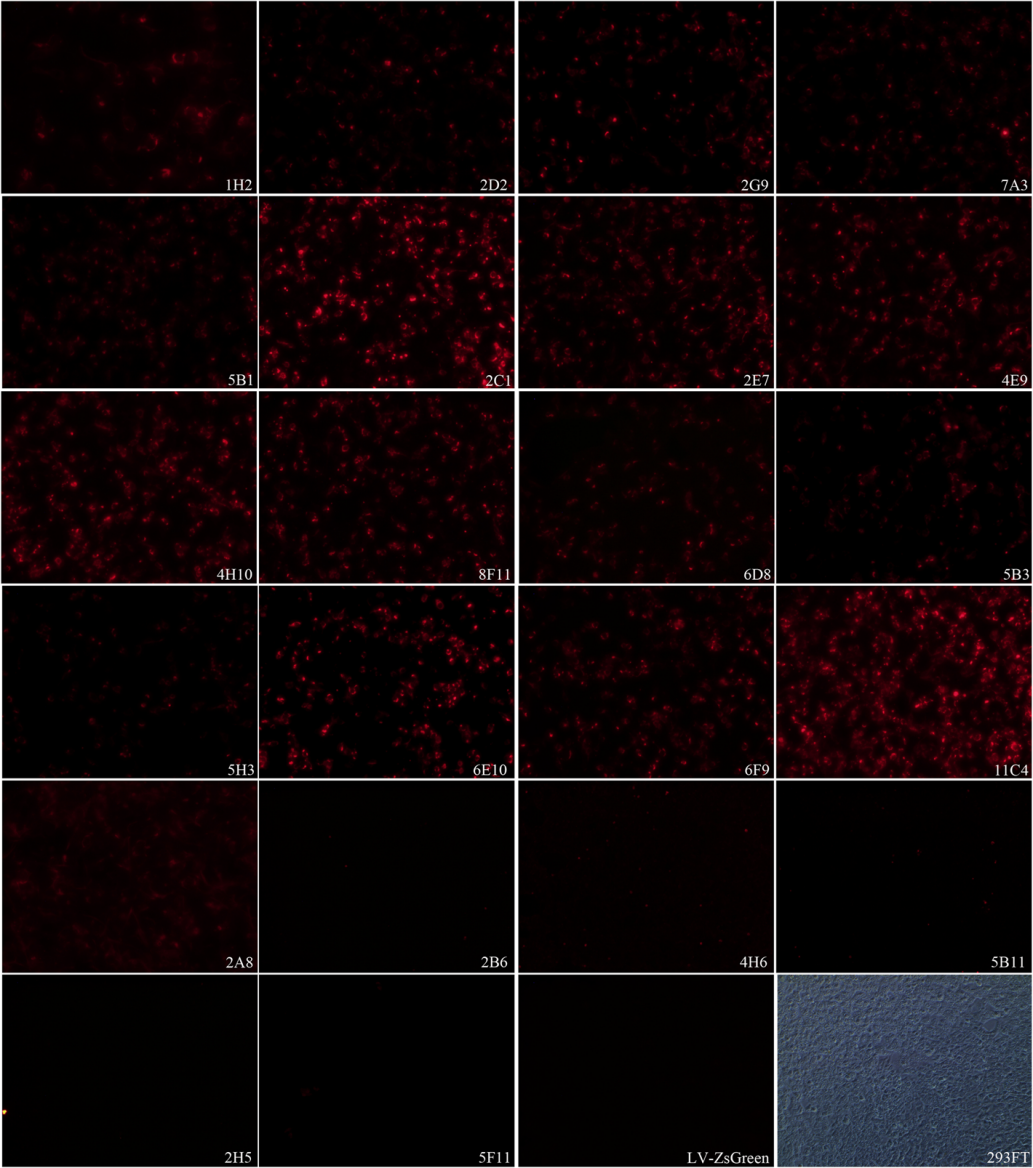
Recognition of lentivirus-mediated Omp31 expressing cells by mAbs in IFS. The lentivirus (LV-HAGE-Omp31) transduced 293FT cells were stained by the IFS with individual mAbs specific to Omp31. Reactivity levels are estimated from immune-stained cells by the fluorescent density of strongly reactive (++), reactive (+) or non-reactive (−), respectively. LV-ZsGreen is a negative control for lentivirus vector transduced cells; 293FT is a control of cells observed in white light under a Nikon Labophot photomicroscope

To detect *Brucella* Omp31 intracellularly, 293FT cells were transduced by recombinant Omp31-lentivirus (LV-HAGE-Omp31) for mimicking *Brucella* infection in human or animal cells. By using IFS, one IgM mAb (2D2) and 16 IgG mAbs were reactive to the expressed rOmp31 in transduced 293FT cells (Fig. 4).

### Identification of *Brucella melitensis* strains by mAbs

**Fig. 5:**
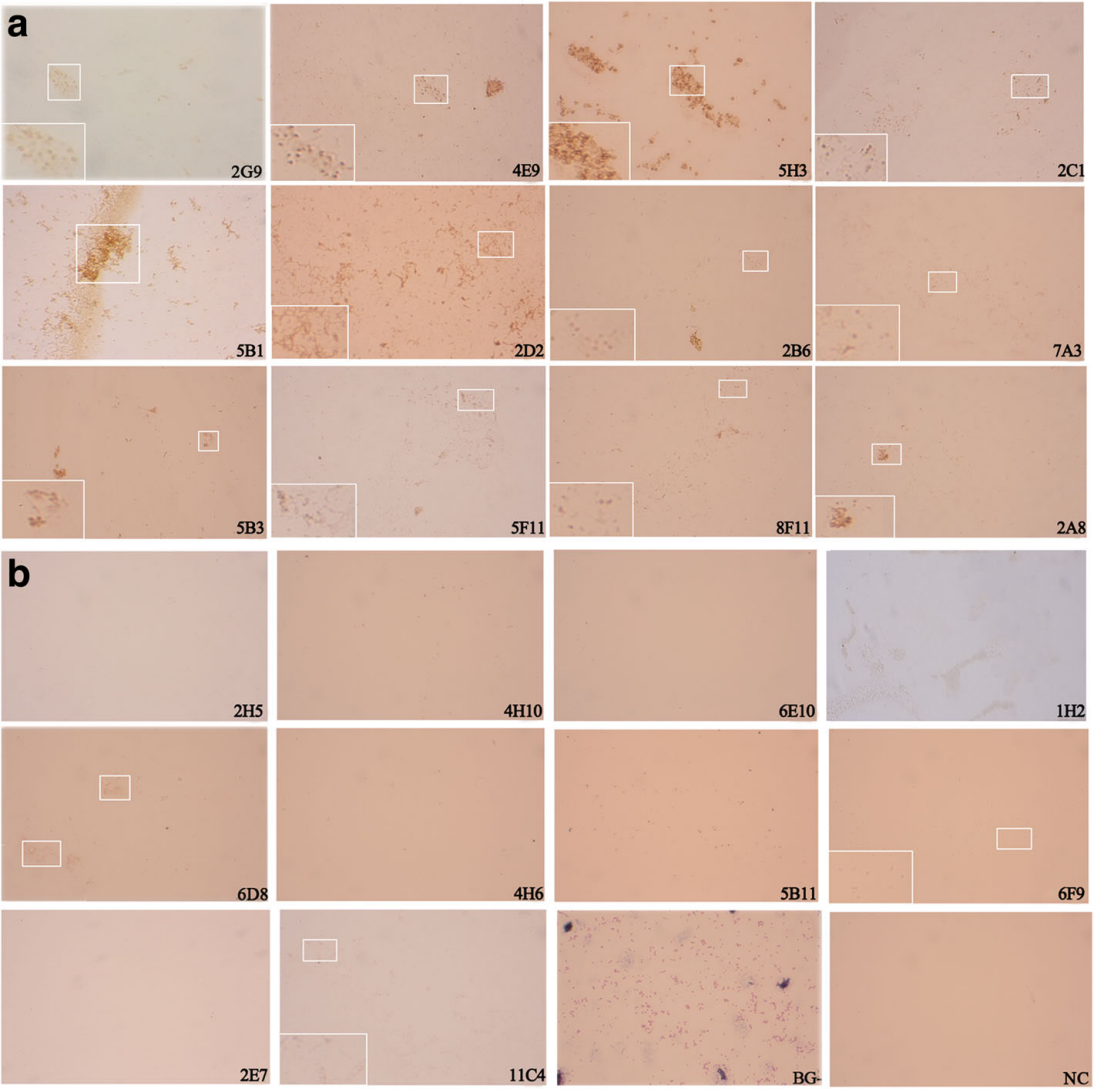
Identification of *B. melitensis* strain by mAbs in ICS. The intact bacteria of *B. melitensis* strain were stained in ICS by individual mAbs specific to Omp31. (**a**) Positive staining; (**b**) Negative, indeterminate or control staining. GB, Gram staining for bacterial control of *B. melitensis* strain viewed in white light under a microbiological microscope. NC = negative control for the ICS with a mAb to HCV NS3

To identify reactivity of mAbs with Omp31 on the membrane of bacteria, the intact *B*. *melitensis* strains were immunologically stained by ICS with mAbs, individually. Of 22 mAbs, 12 were reactive with intact *B*. *melitensis* bacteria by ICS (Fig. 5 and Table 1).

Based on cross-matching reactivity levels of mAbs to the native Omp31 antigen carrying different recognition epitopes, 1 IgG1 (mAb 7A3) and 4 IgG2a (mAbs 5B1, 2C1, 5B3 and 5H3) clones presented high reactive profiles suitable as diagnostic antibodies in immunoassays of Western-blot, ELISA, IFS or ICS (Table 1). Among six IgM clones, mAbs 2D2, 2B6 and 5F11 showed high-level reactivity in EIAs (Table 1), making them more suitable as primary antibodies for capturing the Omp31 antigen in serological tests. In contrast, mAbs 6D8, 2A8 and 4H6 were unable or weakly to react with the native NMP, SSP or intact *B*. *melitensis* strains, suggesting that the recognizing epitopes might not be exposed on the protein surface of Omp31 or *Brucella* strains (Table 1).

## Discussion

Since Omp31 was identified in *B. melitensis* [4], most studies focused on its role in cellular immune protection against *Brucella* infections [7, 8, 10, 12]. Although Omp31 elicited specific antibody production and bactericidal activity [6, 11, 15], it was considered a poor diagnostic antigen [14]. In this study, we prepared 22 murine mAbs against Omp31 of *B. melitensis*, of which 50% (11/22) were IgG2a, 23% (5/22) IgG1 and 27% (6/22) IgM isotypes, respectively. In contrast, we previously found IgG1 to be the majority of isotypes (62% or 18/29) of mAbs to BP26 of *B. melitensis* [16], which might in part explain why BP26 was a diagnostic antigen with higher reactivity to the sera from *Brucella* infected humans or animals. In subclasses of antibodies, mice have IgG1, IgG2a, IgG2b and IgG3, which are functionally similar to human IgG1, IgG2, IgG4 and IgG3, respectively. In general, IgG1 is mainly associated with Th2 but IgG2a with Th1 profiles [17–19]. Therefore, a higher population of IgG2a induced by Omp31 may confer Th1-type immune protection against *Brucella* infection through IFN-γ and up-regulating phagocytosis. However, BP26 mainly induced a major IgG1 subclass antibody and functionally polarized Th2 cells in *Brucella* infection [16–23]. The ratio of IgG isotypes might be varied in relating to different antigenic properties of proteins.

Besides cellular immune response, a specific monoclonal antibody to Omp31 (A59/10F09/G10, IgG2a) was identified previously as a protective factor against *B. melitensis* or *B. ovis* infection in mice [4, 14, 24]. MAb A59/10F09/G10 recognized a hydrophilic loop minimized within aa 48–83 of Omp31, which was exposed on the surface of pathogenic strains of *B. melitensis*, *B. ovis* and *B. canis* [4, 13, 14, 25]. Another study reported that 11 mAbs obtained after immunization with truncated rOmp31 of *B. ovis* recognized epitopes localized at distant positions, such as aa48–83, aa149–182 or aa180–224 [14]. In our study, we depicted characteristics of 22 mAbs, and found that 13 mAbs were reactive with linear epitopes and others with conformational or semi-conformational epitopes [16]. None of the mAbs reacted with the same epitope as mAb A59/10F09/G10. However, we identified five new discreet epitopes (Ep) of Omp31 mapped within 27 16mer overlapping peptides, including Ep5 (aa39–49), Ep11 (aa87–102), Ep20 (aa168–176), Ep21 (aa183–192) and Ep24 (aa204–219) (Fig. 2). It remains to be investigated further whether some of mAbs reacting with these epitopes have the ability to protect animals against *B. melitensis or B. ovis* infection.

In addition, epitopes Ep5 and Ep21 were considered as B-cell epitopes in the present study and shared its aa sequences with Omp31 T-cell epitopes P06 and P21, which were identified in sheep vaccinated with attenuated *B. melitensis* M5–90 [12]. Interestingly, those mAbs recognizing both B and T cell epitopes were all of IgG1 isotype, while other mAbs to B-cell epitopes (Ep11, Ep20 and Ep24) were IgG2a isotype alone (Table 1). A 27 amino acid polypeptide (aa48–74) was previously identified as both T and B cell epitopes inducing cellular and humoral response in mice, and the IgG1 titer was higher than IgG2a in sera [26]. However, it is still unknown whether there are significant discrepancies between IgG1 and IgG2a binding to B or T cell epitopes, or isotypes of mAbs sharing reactivity with different types of epitopes. The mAb A59/10F09/G10 was IgG2a and reactive with a common epitope (aa48–83) of T and B cells [4], this finding being inconsistent with the above description of epitopes mostly associated with IgG1.

A recent study reported a monoclonal antibody-based *B. melitensis* lipopolysaccharide antigen detection by ELISA [27]. In our study, the panel of 22 mAbs to Omp31 was tested to evaluate their reactivity with different forms of recombinant and native Omp31 of *B. melitensis*, including intact bacteria and bacterial or cell extracts detected by Western-blot, ELISA, IFS and ICS. Data in Table 1 showed that 5 IgG and 3 IgM clones of mAbs had suitable ability for the detection of Omp31 or *Brucella* strains by diverse immunoassays. MAbs 7A3, 5B1, 2C1, 5B3 and 5H3 reacted with different Omp31 epitopes exposed on the surface of *B. melitensis* strain and identified by ICS. Few substitutions were found in alignments of Omp31 aa sequences of *B. melitensis*, *B. ovis*, *B. suis* and *B canis* strains. The five new epitopes (Ep5, Ep11, Ep20, Ep21 and Ep24) identified in this study had conserved sequences except for a single aa mutation within Ep20 (Fig. 3), suggesting that these mAbs could be used to detect at least four species of *Brucella* strains. The IFS detection of intracellular *Brucellae* might be an alternative assay of conventional bacterial culture for examination of *Brucella* elimination by brucellosis treatment in clinical practice [28].

## Conclusions

This study identified 22 novel mAbs specific to Omp31 of *B. melitensis*. Five IgG and 3 IgM clones presented high ability to recognize multiple epitopes of Omp31 antigen, which were exposed on the protein surface of intact *B. melitensis* and highly conserved among *B. melitensis*, *B. ovis, B. suis* and *B. canis* strains. The monoclonal antibodies obtained in this study could provide a substantial help as key reagents in diagnostic tools for identifying *Brucella* Omp31 antigens in laboratory, or for detecting intracellular *Brucella melitensis* and other *Brucella* species beyond *B. abortus* in clinical.

## Methods

### Animals

Mice were obtained from the Animal Experimental Center of Southern Medical University (SMU), Guangzhou, China. Animal care was in accordance with national and institutional policies for animal health and well-being. Mouse surgery was performed under anesthesia for minimizing suffering of animals.

### Recombinant Omp31 (rOmp31)

The full-length gene of 221 amino acids (aa) encoding for the matured Omp31 (excluding 19 aa of signal polypeptides) from attenuated vaccine strain of *B. melitensis* M5–90 was constructed with pET-30a plasmid (pET-Omp31) and expressed in *E.coli* as described previously [15, 16]. The soluble recombinant Omp31 was obtained at 95% purity and used for mouse immunization and serological tests.

### Monoclonal antibody production

The 6-week old BALB/c female mice were immunized with three injections of rOmp31 antigen at 2-week intervals as previously described [15, 16]. The spleen cells prepared from the immunized mice were fused with SP2/0 myeloma cells by PEG 4000 (Sigma-Aldrich, St Louis, Missouri, United States). The hybridoma cells secreting monoclonal antibodies (mAbs) to rOmp31 were individually selected up to a single clone by EIA [15, 16]. All clones were passaged in producing cells for a period of 6 months and kept frozen in liquid nitrogen after which antibodies were kept frozen at −20 °C. Pre-immunization and immunized sera were collected and used as the negative or positive controls for screening mAbs, respectively. One mAb (IgG1 kappa) to recombinant non-structural protein-3 (NS3) of hepatitis C virus (HCV) was used as an unrelated negative control [29]. MAb isotyping was performed by IsoQuick Strips (Sigma-Aldrich, St Louis, Missouri, United States).

### Lentivirus-mediated Omp31 expression ex vivo

Omp31 gene was transferred into a lentiviral vector and packaged as an infectious recombinant lentivirus LV-HAGE-Omp31. Omp31 was expressed in 293FT cells by Omp31-lentivirus-mediated transduction as described previously [30], mimicking Omp31 antigen in *Brucella*-infected mammalian cells.

### Immunoassays

Recombinant Omp31 and native Omp31-containing membrane protein extracts (NMP) from *B. melitensis* M5–90 were used in ELISA for mAbs detection [15]. A panel of 27 16mer overlapping peptides spanning full-length of Omp31 sequence were applied in peptide-ELISA to identify epitopes recognized by mAbs [12]. Cutoffs were calculated as mean OD + 2SD with 95% confidence interval (CI) of three negative controls. Western-blot was used to identify the reactivity of mAbs with rOmp31 or NMP extracted from transformed *E. coli*, *B. melitensis* or transduced cells [12, 30]. Lentivirus LV-HAGE-Omp31 transduced cells were detected by immunofluorescent staining (IFS) with individual mAbs. The intact *B. melitensis* strains were examined by immunochemical staining (ICS) under a microbiological optical microscope (Olympus, Japan).

A goat anti-mouse IgG and IgM horseradish peroxidase (HRP)-conjugate (Rockland Immunochemicals Corp, Boyertown, Pennsylvania, USA) was used as secondary antibody in ELISA and ICS. DyLightTM594-conjugated AffiniPure Goat Anti-Mouse IgG + IgM (H + L) (Jackson ImmunoResearch Laboratories, Inc., West Grove, PA, USA) was used as secondary antibody in IFS. A mAb to HCV NS3 (IgG1) was used as negative control.

## Abbreviations

*B.*: *Brucella* spp.
EIA: Enzyme immunoassay
ELISA: Enzyme linked immunosorbent assay
ICS: Immunochemical staining
IFS: Immunofluorescent staining
mAb: Monoclonal antibody
Omp31: Outer membrane protein 31
